# Whole exome sequencing identified a novel *POT1* variant as a candidate pathogenic allele underlying a Li–Fraumeni-like family

**DOI:** 10.3389/fonc.2022.963364

**Published:** 2022-11-01

**Authors:** Yuping Li, Yupeng Xie, Di Wang, Hanyan Xu, Junru Ye, Jiani C. Yin, Junjie Chen, Junrong Yan, Bin Ye, Chengshui Chen

**Affiliations:** ^1^ Key Laboratory of Interventional Pulmonology of Zhejiang Province, The First Affiliated Hospital of Wenzhou Medical University, Wenzhou, Zhejiang, China; ^2^ Department of Pulmonary and Critical Care Medicine, The First Affiliated Hospital of Wenzhou Medical University, Wenzhou, Zhejiang, China; ^3^ Medical Department, Nanjing Geneseeq Technology Inc., Nanjing, Jiangsu, China

**Keywords:** Li-Fraumeni-like syndrome, whole exome sequencing (WES), co-segregation, genetic risk alleles, POT1

## Abstract

**Background:**

Li-Fraumeni syndrome (LFS) and Li-Fraumeni-like (LFL) syndrome are rare hereditary diseases characterized by predisposition to a diverse spectrum of cancer types, primarily sarcoma. The pathogenic variants underlying the majority of LFL cases remain to be explored.

**Methods:**

We performed whole-exome sequencing (WES) on 13 core members of a large LFL family with highly aggregated incidences of cancers, including cases with sarcoma, non-small cell lung cancer and cardiac angiosarcoma, and conducted a comprehensive literature review of candidate gene associations in LFS/LFL syndromes or sarcoma to identify potential pathogenic germline variants.

**Results:**

No germline variants in the best-known LFL/LFS-associated gene *TP53* were detected. Of all the genes associated with LFS/LFL or sarcoma that we have surveyed, we identified a novel p.P35L germline variant in *POT1* (protection of telomeres 1). Germline and somatic alterations in *POT1* have been implicated in a series of familial cancers, including angiosarcoma, glioma, melanoma and colorectal cancer. This particular variant is located in the telomere-binding OB1 domain, which is important in maintaining the proper telomere length, and showed high conservation across different POT1 orthologues. No record of the variant was found in any of the 1000 genomes, ExAC, gnomAD, dpSNP and COSMIC databases. Prediction algorithms and *in silico* structural analysis suggested completely disrupted protein structure and function of POT1 in the presence of this mutation.

**Conclusions:**

Leveraging WES, we identified a novel germline risk allele, p.P35L in *POT1*, that likely predisposes to LFL syndrome. Our results support the routine testing of *POT1* and other LFL/LFS-associated genes in the risk populations to enable early cancer diagnosis, prevention and intervention.

## Introduction

Li-Fraumeni syndrome (LFS) and Li-Fraumeni-like (LFL) syndrome are clinically heterogeneous, autosomal dominant disorders (1), which are characterized by increased risks of early-onset cancers that may arise from multiple organ systems. The common cancer types associated with LFS/LFL are breast cancer, soft-tissue sarcoma, brain tumors, osteosarcoma and hematological malignancies (2). In addition, a wide range of cancer types, including lung, gastric, colorectal, liver, and pancreas cancers, among others, have been reported at a relatively low incidence in families with LFS/LFL (3). The classical criteria for LFS are a proband with sarcoma diagnosed before the age of 45 and a first-degree relative diagnosed with cancer before the age of 45, plus a first- or second-degree relative with cancer before the age of 45 or sarcoma at any age (3). The classification criteria for LFL syndrome are less stringent.

The best-characterized LFS/LFL gene is *TP53*, of which germline mutations can be found in approximately 70% of LFS and 20-40% of LFL families (3, 4). Carriers of germline *TP53* mutations have a much higher risk for cancers at a young age (5), with an estimated lifetime risk of 70% in males and nearly 100% in females due to the high incidence of breast cancer (3, 6). The remaining *TP53*-negative LFS/LFL families indicate the presence of additional susceptibility genes. *CHEK2* encodes a cell cycle checkpoint regulator that participates in the processes of DNA repair, cell death, and cell cycle control through stabilization of p53 (7). While loss of function *CHEK2* mutations might contribute to increased risks of individual cancer development in LFS/LFL families, several studies have ruled out *CHEK2* as a major LFS/LFL susceptibility gene (8, 9). However, due to the low incidence of LFS/LFL and limited tumor testing in the affected families, the causative alleles of the remaining LFS/LFL families are yet to be identified.

In this study, we report a LFL family with early-onset sarcoma and lung cancers. Whole exome sequencing (WES) was performed on core family members to identify candidate risk variants. Associations with cancer development and functional predictions of pathogenicity were performed to uncover potential pathogenic variants.

## Method

### Study subjects and sample collection

**Figure 1:**
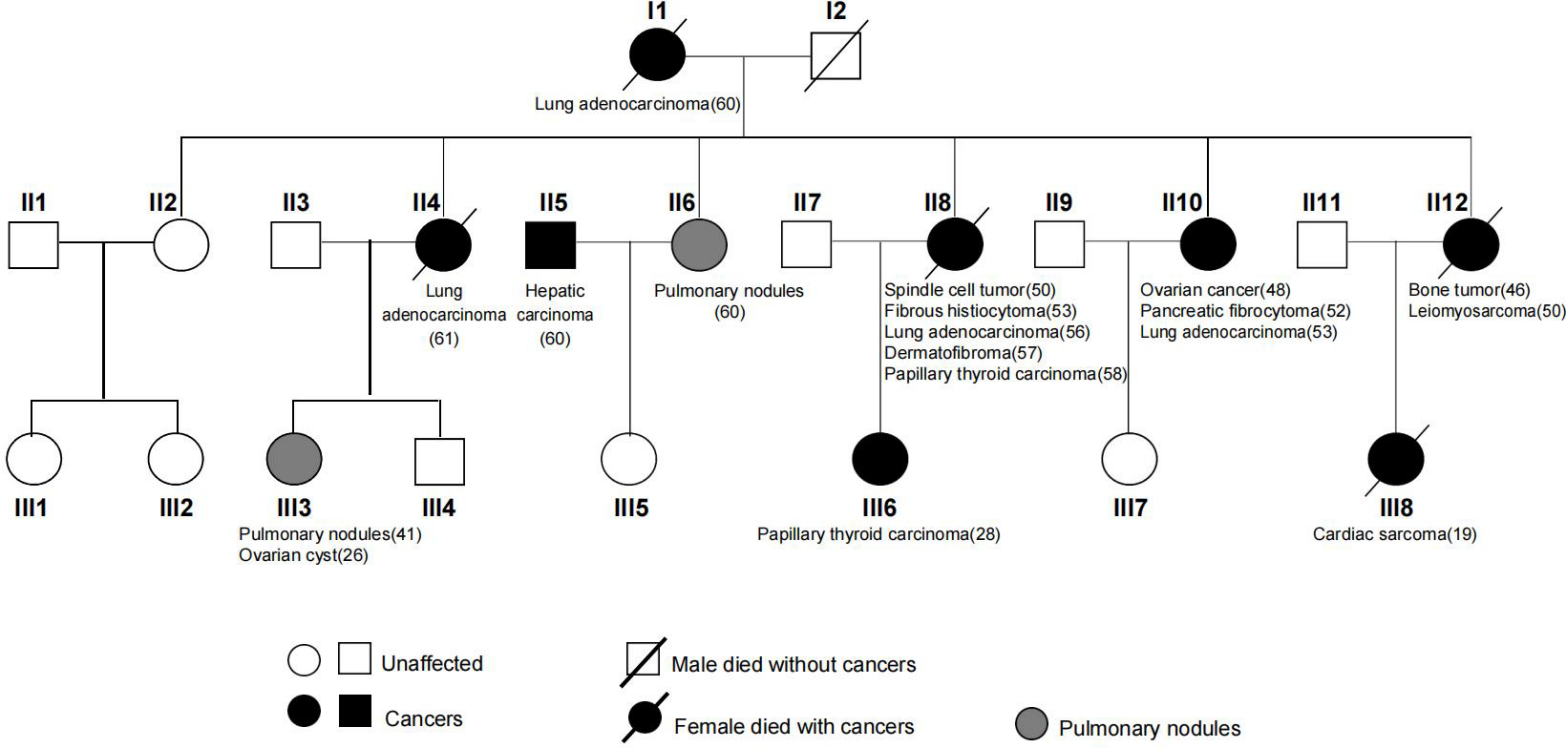
Pedigree of the LFL family across three generations in this study.

A Chinese LFL family of multiple affected individuals with sarcoma and NSCLCs were studied (**Figure 1**). WES was performed on 13 core members of the second and third generations. A comprehensive search of the Pubmed database was performed by using terms related to LFS, LFL, sarcoma, susceptibility variants, and familial cancer syndromes. Manual review of the relevant articles was conducted. Written informed consents were obtained from all family members. The study was approved by the Ethics Committee of the First Affiliated Hospital of WenZhou Medical University and was conducted in accordance with the Declaration of Helsinki.

### Exome sequencing

Sequencing tests were performed in a CLIA-certified and CAP-accredited NGS testing center (Nanjing Geneseeq Technology Inc., China). In brief, genomic DNA from whole blood and tumor tissues were extracted by using the DNeasy Blood and Tissue kit (Qiagen). Purified genomic DNA was qualified by Nanodrop2000 for A260/280 and A260/A230 ratios (Thermo Fisher Scientific). Fragmented DNA was subjected to library preparations using KAPA Hyper Prep Kit (KAPA Biosystems). Exome capture was performed using the xGen Exome Research Panel v2 (Integrated DNA Technologies) and Hybridization and Wash Reagents Kit according to manufacturer’s protocol. Sequencing was performed with enriched libraries on an Illumina HiSeq 4000 platform. The mean coverage depths were ~68X for the white cell controls and ~137X for the tumor tissue.

### Mutation calling

Trimmomatic was used for FASTQ file quality control. Sequencing reads with low quality (quality reading below 20) or N bases were removed. Paired-end reads were then aligned to the reference human genome (build hg19), using the Burrows-Wheeler Aligner (BWA, https://github.com/lh3/bwa/tree/master/bwakit) with default parameters. PCR deduplication was performed using Picard (https://broadinstitute.github.io/picard/) and local realignment around indels and base quality score recalibration were performed using GATK3 (https://software.broadinstitute.org/gatk/). The GATK Haplotype caller was used for germline variant calling. Single-nucleotide variations (SNVs) and insertion/deletion mutations were detected using VarScan2 and Mutect2, with a minimum variant allele frequency threshold set at 0.01 and p-value threshold for calling variants set at 0.05 to generate Variant Call Format files. All SNVs/indels were annotated with ANNOVAR, and each SNV/indel was manually checked on the Integrative Genomics Viewer. Common SNPs with more than 1% of population frequency in the 1000 Genomes Project or the Exome Aggregation Consortium (ExAC) exome database were excluded. Copy number ratios were calculated by CNVKit (https://cnvkit.readthedocs.io). *In silico* structural modeling was performed by using PyMOL (pymol.org).

## Result

### History and clinical investigation of the LFL family

A 61-year-old female patient (II4) was admitted to our hospital and diagnosed with lung adenocarcinoma. Detailed clinical consultation revealed a strong family history of cancer diagnosed at relatively young ages, suggesting that cancer development might be hereditary. A complete family history across three generations was obtained, with the pedigree shown in **Figure 1**. Clinical characteristics of the patients are summarized in **Table 1**. The consultand’s mother was diagnosed with lung adenocarcinoma at the age of 60. In addition, three sisters (II8, II10, and II12) were also diagnosed with cancers at young ages. Of the three, two sisters (II8 and II10) were first diagnosed with spindle cell tumor and fibrous histiocytomas of the pancreas at 50 and 52 years of age, respectively. Both of them subsequently developed lung adenocarcinoma at ages 56 and 53, respectively. Moreover, II8 developed with bilateral papillary thyroid carcinoma at the age of 58. Another sister (II12) was diagnosed with bone cancer at age 46 and later with leiomyosarcoma at age 50. Two other sisters were otherwise unaffected, although one of them (II6) was detected with multiple pulmonary nodules at age 60. The consultand’s daughter (III3) was detected with pulmonary nodules and ovarian cysts at age 41 and 26, respectively. Extended family history was taken and we found two third-generation females (III6 and III8) with cancer histories. Notably, III8, the daughter of II12, had been diagnosed with rare early-onset cardiac angiosarcoma (CAS) and died from the disease at age 19. III6, the daughter of II8, were diagnosed with papillary thyroid carcinoma at the age of 28. All of the above tumors diagnosed in this kindred have been confirmed as primary lesions. At the time of manuscript preparation, II4, II8 and II12 died at ages of 65, 59 and 52 respectively. Taken into account the family histories, cancer types and age of onset, we considered the kindred to fit the LFL classification criteria.

**Table 1:** Clinical characteristics of individuals in the LFL family.

Patient ID	Sex	Deceased	Age of onset	Diagnosis	Smoking
II2	Female	N	/	Unaffected	NO
II4	Female	Y: Age of 65	61	Lung adenocarcinoma	NO
II6	Female	N	60	Pulmonary nodules	NO
II8	Female	Y: Age of 59	50	Spindle cell tumor	NO
			5	Fibrous histiocytoma	
			56	Lung adenocarcinoma	
			57	Dermatofibroma	
			58	Papillary thyroid carcinoma	
II10	Female	N	48	Ovarian cancer	NO
			52	Pancreatic fibrocytoma	
			53	Lung adenocarcinoma	
II12	Female	Y: Age of 52	46	Bone tumor	NO
			50	Leiomyosarcoma	
III1	Female	N	/	Unaffected	NO
III3	Female	N	41	Pulmonary nodules	NO
			/	Ovarian cyst	
III4	Male	N	/	Unaffected	NO
III5	Female	N	/	Unaffected	NO
III6	Female	N	28	Papillary thyroid carcinoma	NO
III7	Female	N	/	Unaffected	NO
III8	Female	Y: Age of 19	19	Cardiac angiosarcoma	NO

### Identification of candidate LFL variants

To identify candidate pathogenic germline variants in this LFL family, WES was performed on core family members, including six cancer patients (II4/8/10/12 and III6/8), as well as seven unaffected individuals (II2/6 and III1/3/4/5/7). To search for potential causative alleles, we assessed germline variants with frequency of less than 1% in the general or the East Asian populations based on the gnomAD_exome database. As LFS and LFL syndromes can be ascribed mostly to *TP53* mutations, we first checked for the presence of *TP53* variants in association with cancer development. However, no germline variants in *TP53* were detected in any of the individuals. Next, we performed literature review on LFS, LFL or sarcoma-relevant susceptibility genes and narrowed down our search to 98 genes that might explain the increased cancer risks in our LFL family (**Table 2**).

**Table 2:** List of candidate genes associated with LFS/LFL and sarcoma.

Disease	Gene Name
LFS/LFL	*TP53 (* 3 *), CHEK2 (* 8 *), POT1 (* 10, 11 *), BRCA2 (* 12 *), IDH1 (* 13 *), ATRX (* 14 *), MDM2 (* 15 *)*
Sarcoma	*ABCB5 (* 16 *), APC (* 17 *), AR (* 18 *), ATM (* 19 *), ATRX (* 17 *), ATR (* 19 *), BAG1 (* 20 *), BRCA1 (* 19 *), BRCA2 (* 19 *), BRIP1 (* 19 *), BUB1B (* 21 *), C16orf96 (* 16 *), CD86 (* 22 *), CD99 (* 23 *), CDKAIC (* 21 *), CDKN2A (* 17 *), CHEK2 (* 19 *), CMMRD (* 23 *), COL7A1 (* 19 *), CREBBP (* 21 *), CTLA-4 (* 24 *), DICER1 (* 19 *), EGR2 (* 23 *), ERCC2 (* 19 *), EWS (* 23 *), EXT1 (* 21 *), EXT2 (* 21 *), FAH (* 17 *), FANCC (* 19 *), FANCI (* 19 *), FANCL (* 19 *), FANCM (* 25 *), Fanconi Anemia genes mutations (* 23 *), FAS (* 26 *), FGF2 (* 25 *), FGFR3 (* 25 *), FH (* 21 *), GATA1 (* 27 *), GH1 (* 25 *), GJB2 (* 19 *), GNRH2 (* 25 *), GRM4 (* 28 *), GSTM1 (* 29 *), GSTT1 (* 29 *), H19 (* 21 *), HRAS (* 21 *), IDH1 (* 13 *), IGF1 (* 25 *), IGF2 (* 21 *), IGFALS (* 18 *), ITGA3 (* 30 *), KCNQ1OT1 (* 21 *), KIT (* 21 *), LIT1 (* 21 *), LOX (* 31 *), LZTR1 (* 19 *), MDM2 (* 32 *), MEN1 (* 17 *), MPG (* 25 *), MSH2 (* 17 *), MSH6 (* 19 *), MUTYH (* 19 *), NF1 (* 21 *), NFIB (* 33 *), NBS1 (* 21 *), NPAS2 (* 34 *), PALB2 (* 19 *), PDGFRA (* 21 *), PMS2 (* 19 *), POLE (* 19 *), POT1 (* 17 *), PTCH (* 21 *), PTEN (* 35 *), PTPN11 (* 21 *), PTPRD (* 19 *), RB1 (* 27 *), RECQL2 (* 27 *), RECQL3 (* 27 *), RECQL4 (* 27 *), RECQL5 (* 36 *), RET (* 19 *), ribosomal protein genes (* 27 *), SDD (* 21 *), SDHB (* 21 *), SDHC (* 21 *), SMARCB1 (* 21 *), SOS1 (* 21 *), SQSTM1 (* 37 *), TERF1 (* 38 *), TGFBR1 (* 39 *), TNF-α (* 40 *), TP53 (* 27 *), TSC1 (* 21 *), TSC2 (* 21 *), TSC3 (* 21 *), VEGF (* 41 *), VHL (* 17 *)*

**Figure 2:**
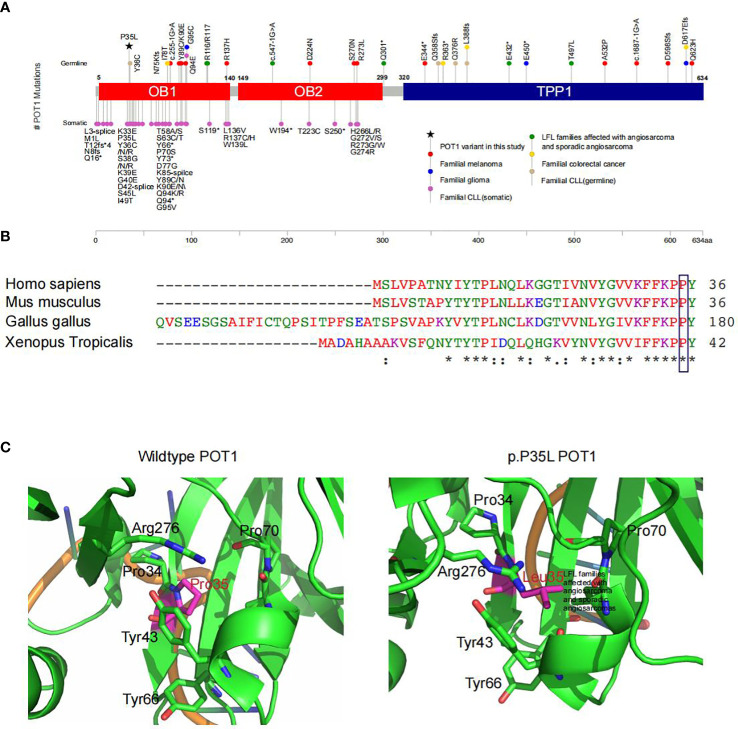
Functional prediction of the *POT1* variant in association with LFL. **(A)** Summary of reported *POT1* variants. Germline and somatic variants in *POT1* underlie a series of familial cancers, including angiosarcoma (green), glioma (blue), melanoma (red), colorectal cancer (yellow) and chronic lymphocytic leukemia (pink and light brown) as indicated. The *POT1* p.P35L variant detected in this pedigree is marked with a black asterisk, which is located in one of the OB fold domains. **(B)** Sequence alignment showing strong conservation of the POT1 p.P35 residue across different orthologues. An “*” (asterisk) indicates positions which have a single, fully conserved residue. A “:” (colon) indicates conservation between groups of strongly similar properties. A “.” (period) indicates conservation between groups of weakly similar properties. **(C)** *In silico* structural modeling of the POT1 protein indicated change of the proline residue at position 35 to leucine led to significant conformational change of the protein, which would affect binding of the telomeres.

Interestingly, we detected a p.P35L variant in protection of telomeres (*POT1*). The *POT1* p.P35L variant was detected in all of the affected individuals with cancers, including those with NSCLC, sarcoma, thyroid cancers, as well as the individual with childhood-onset cardiac angiosarcoma (III8) (**Table 3**). Germline and somatic mutations of *POT1* have been reported to underlie a series of familial cancers, including angiosarcoma, glioma, melanoma and colorectal cancer (10, 42). *POT1* is also frequently mutated in chronic lymphocytic leukemia (43) (**Figure 2A**). Notably, a p.R117C variant in *POT1* has been reported in *TP53*-negative LFL families with cardiac and breast angiosarcoma (11). On the other hand, the p.P35L variant in our reported family has not been described in the literature or any of the existing databases, including the 1000 Genomes, ExAC, gnomAD, dpSNP and COSMIC databases, which is in accordance with the American College of Medical Genetics (ACMG) guidelines with moderate evidence for pathogenicity (PM2 evidence).

**Table 3:** List of *POT1* associated with cancer development in the LFL family.

Gene Name	Variant	Mutant Type	Gerp++ RS	SIFT_ score	SIFT_ prediction	Polyphen_score	Polyphen_prediction	Carrier	Non-carrier
*POT1*	c.104C>T (p.P35L)	Missense	5.71	0.01	D	1	D	III4/8/10/12 III6/8	III2/6 III1/3/4/5/7

*POT1* encodes a core component of the shelterin complex, which functions to bind and protect the telomeres (44). The p.P35 residue is located in the one of the oligonucleotide/oligosaccharide binding (OB) fold domains that directly bind the telomeres (42) (**Figure 2A**). Comparisons of the POT1 amino acid sequences across different orthologues showed strong conservation of the p.P35 residue (**Figure 2B**). Indeed, alteration of the p.P35 residue was predicted to be deleterious to protein function by all existing algorithms such as SIFT (score=0.01), Polyphen (score=1) and GERP (score=5.71) (**Table 3**), which is consistent with PP3 evidence of the ACMG guidelines. Importantly, change of the proline residue at position 35 to leucine led to significant conformational change of the protein, and consequently would affect binding of the telomeres (**Figure 2C**).

## Discussion

Given their low incidence and highly heterogeneous nature, the genetic mechanisms underlying *TP53*-negative LFS and LFL syndromes have not been extensively studied. In this study, we report a Chinese family with LFL syndrome, consisting of individuals with lung cancers, thyroid cancers and a case with highly rare cardiac angiosarcoma, all occurred at relatively young ages. WES profiling of core family members and comprehensive literature review revealed a potentially causative missense variant (p.P35L) in *POT1.* The p.P35 residue lies in the telomere binding domain and is highly conserved across different orthologues. Pathogenicity of the variant is further supported by functional prediction algorithms and *in silico* structural modeling analysis.

Previous studies have demonstrated that individuals with germline *TP53* variants have significantly increased risks of a broad range of neoplasms, involving the lung, stomach, skin and gonadal germ cells (45). In over 70% LFS cases, cancer is driven by mutations in the *TP53* gene. On the other hand, only 20-40% of LFL syndrome families can be accounted for by the presence of *TP53* germline mutation (3, 4). Thus, additional susceptibility genes remain to be identified in approximately 30-60% of LFS/LFL syndromes. It has been reported that alterations in the cell cycle checkpoint kinase, *CHEK2*, might be associated with increased cancer risks in LFS/LFL families (8). However, *CHEK2* has been excluded as a major LFS gene, but rather represents a moderate risk gene for individual cancer susceptibility within LFS clusters (46, 47). In this kindred, no germline variants in *TP53* and *CHEK2* were detected (**Supplementary Table 1**). It is worth noting that exon 1 of *TP53* were not fully covered by the WES panel used in this study, so we cannot exclude the possibility that this family might also carry loss-of-function mutations in *TP53* in addition to *POT1* mutations. On the other hand, we performed additional genetic analysis on the available tumors to check for the presence of *TP53*, *CHEK2* and *POT1*. WES was performed on tumor tissue samples from four patients (II4/8/10/12), consisting of lung tumor samples from II4/8/10 and a leiomyosarcoma sample from II12. Two somatic variants in *TP53*, p.D259Y and p.S215R, were detected in II10 and II12, respectively. The *TP53* p.D259Y is well-defined pathogenic that can reduce the tumor suppressor function of p53, while the p.S215R alteration is of unknown significance. In addition, driven gene mutations were found in two of the patients, including an *EGFR* p.L858R in II10 and a *KRAS* p.G12D in II12. However, no additional POT1 variants were found in any of the tumor samples (**Supplementary Table 2**).

One previous study has described a p.R117C variant in *POT1*, which results in unstable telomeres, as a causative allele of cardiac angiosarcoma in LFL families (11). Further study by the same group has uncovered a wide spectrum of *POT1* variants in patients with familial angiosarcoma, as well as sporadic angiosarcoma and sarcomas (10). A considerable proportion of reported *POT1* mutations in familial cancer syndromes occur in the telomere-binding OB1 domain (**Figure 2A**). The novel p.P35L variant identified in our kindred is also located in the OB1 domain with potential deleterious effects to protein structure and function. Subsequent characterization of the spectrum of *POT1* mutations in 62,368 solid tumors has revealed a strong association between the presence of *POT1* mutations and angiosarcoma development (10). In line with this study, one of the *POT1* variant carriers, the III8 patient in our LFL family, presented with cardiac angiosarcoma and died at an early age of 19. Angiosarcomas, which commonly occur in the skin, breast, liver and deep tissues, are rare malignancies with highly aggressive nature (48). Angiosarcomas of the heart are extremely rare and associated with high recurrence and metastasis rates, with an estimated frequency of 0.001% to 0.003% in the general population (49). Given the rarity of the disease and unspecific symptoms, such as shortness of breath, weight loss and fatigue, early diagnosis can be very challenging (48). Screening for *POT1* and other LFS/LFL or angiosarcoma causative genes might be of great clinical relevance. It is worth noting that no *POT1* mutations were detected in the two individuals (II6 and III3) with pulmonary nodules and/or ovarian cysts. As smoking status and environmental exposure might be related to the development of pulmonary nodules, it had been first confirmed that both II6 and III3 are non-smokers with no obvious environmental exposures. II6’s husband was diagnosed with hepatocellular carcinoma at the age of 60, although it is unclear whether this has anything to do with the family’s living habits. Moreover, the two patients and their family members have no history of tuberculosis infection, as tuberculosis infection may be one of the factors causing pulmonary nodules. On the other hand, it should be noted that the incidence of pulmonary nodules is extremely high (~ 50%) in China. Given the large number of individuals with pulmonary nodules and the absence of clear environmental causes, the two cases with pulmonary nodules may be accidental events and are likely unrelated to the hereditary condition in this LFL family. Meanwhile, long-term monitoring of all the unaffected individuals in this family might be necessary given the age-dependent penetrance of hereditary cancer syndromes. In addition, we noticed that three NSCLC patients whom were detected *POT1* p.P35L variant are all non-smokers. Of the 13 individuals included in our study, 12 were females, including the three *POT1*-positive non-smoker patients with NSCLC. This is not surprising as a very low percentage of Chinese females are smokers. The fact that these patients had developed lung cancer is consistent with our hypothesis that genetic alterations, rather than environmental exposures, underlie pathogenesis in our patients. We think that the presence of this variant in non-smokers with lung cancer is interesting and further supports our finding that *POT1* alteration is the driving event in our patients.

The molecular basis of most familial hereditary cancer syndromes remains elusive. Leveraging high throughput next-generation sequencing, our study, together with other published work (3, 50), has identified a novel causative variant in a LFL family that provide insight into the pathogenesis of hereditary cancer syndromes and might facilitate early prevention of the diseases. While further validation studies might be necessary to firmly establish the causal relationship between the *POT1* p.P35L variant and multi-cancer phenotype, given the general relevance of *POT1* in LFS/LFL and angiosarcoma patients, our study support the incorporation of *POT1* testing into cancer gene panels for routine diagnostic purposes to enable early diagnosis, prevention and therapeutic intervention of individuals at risk.

## Data availability statement

The datasets presented in this study can be found in online repositories. The names of the repository/repositories and accession number(s) can be found below: The Genome Sequence Archive for Human (GSA-Human), The National Genomics Data Center, HRA001931 at https://ngdc.cncb.ac.cn/search/?dbId=hra&q=HRA001931&page=1.

## Ethics statement

The study was approved by the Ethics Committee (protocol number is KY2021-156d) of the First Affiliated Hospital of WenZhou Medical University and was conducted in accordance with the Declaration of Helsinki. The patients/participants provided their written informed consent to participate in this study. Written informed consent was obtained from the individual(s) for the publication of any potentially identifiable images or data included in this article.

## Author contributions

YL and CC involved in conception and design. HX, JC and JuY carried out provision of study material or patients. DW, JCY, JRY and BY interpreted the data. DW and JRY provided NGS technical support. YL and YX wrote the manuscript. All authors contributed to the article and approved the submitted version.

## Acknowledgments

We gratefully thank the patients and their families who gave consent on presenting the data in this study.

## Conflict of interest

DW, JCY, JRY and BY are employees of Nanjing Geneseeq Technology Inc.

The remaining authors declare that the research was conducted in the absence of any commercial or financial relationships that could be constructed as a potential conflict of interest.

## Publisher’s note

All claims expressed in this article are solely those of the authors and do not necessarily represent those of their affiliated organizations, or those of the publisher, the editors and the reviewers. Any product that may be evaluated in this article, or claim that may be made by its manufacturer, is not guaranteed or endorsed by the publisher.

## Supplementary material

Supplementary Table 1 All germline variants detected in the family members.

Supplementary Table 2 All somatic variants detected in four second-generation patients diagnosed with cancer.
